# Waterfowl Conservation in the US Prairie Pothole Region: Confronting the Complexities of Climate Change

**DOI:** 10.1371/journal.pone.0100034

**Published:** 2014-06-17

**Authors:** Neal D. Niemuth, Kathleen K. Fleming, Ronald E. Reynolds

**Affiliations:** 1 United States Fish and Wildlife Service Habitat and Population Evaluation Team, Bismarck, North Dakota, United States of America; 2 United States Fish and Wildlife Service Division of Migratory Bird Management, Laurel, Maryland, United States of America; DOE Pacific Northwest National Laboratory, United States of America

## Abstract

The Prairie Pothole Region (PPR) is the most important waterfowl production area in North America. However, waterfowl populations there are predicted to decline because of climate-related drying of wetlands. Consequently, changes in the geographic focus of PPR waterfowl conservation have been recommended, which could have long-lasting and costly impacts. We used a 40-year dataset of pond counts collected in the PPR to test hypotheses about climate-related drying. We assessed May (1974–2013) and July (1974–2003) pond numbers in 20 waterfowl survey strata to determine if trends in pond numbers were consistent with predictions of drying. We also assessed trends in precipitation and temperature for the 20 strata and developed models describing May pond numbers from 1974 through 2010 as a function of precipitation, temperature, the previous year’s pond numbers, and location. None of the 20 strata showed significant declines in May pond numbers, although seven strata showed increases over time. July pond numbers declined significantly in one stratum, and increased in seven strata. An index to hydroperiod showed significant increasing trends in three strata, and no strata had decreasing trends. Precipitation increased significantly in two strata and decreased in two from 1974 to 2010; no strata showed significant changes in temperature. The best linear model described pond numbers within all strata as a function of precipitation, temperature, the previous year’s pond numbers, and the latitude and longitude of the stratum, and explained 62% of annual variation in pond numbers. We hypothesize that direct effects of climate change on prairie pothole wetlands and waterfowl may be overshadowed by indirect effects such as intensified land use and increased pressure to drain wetlands. We recommend that an adaptive, data-driven approach be used to resolve uncertainties regarding direct and indirect effects of climate change on prairie wetlands and waterfowl, and guide future conservation efforts.

## Introduction

**Figure 1:**
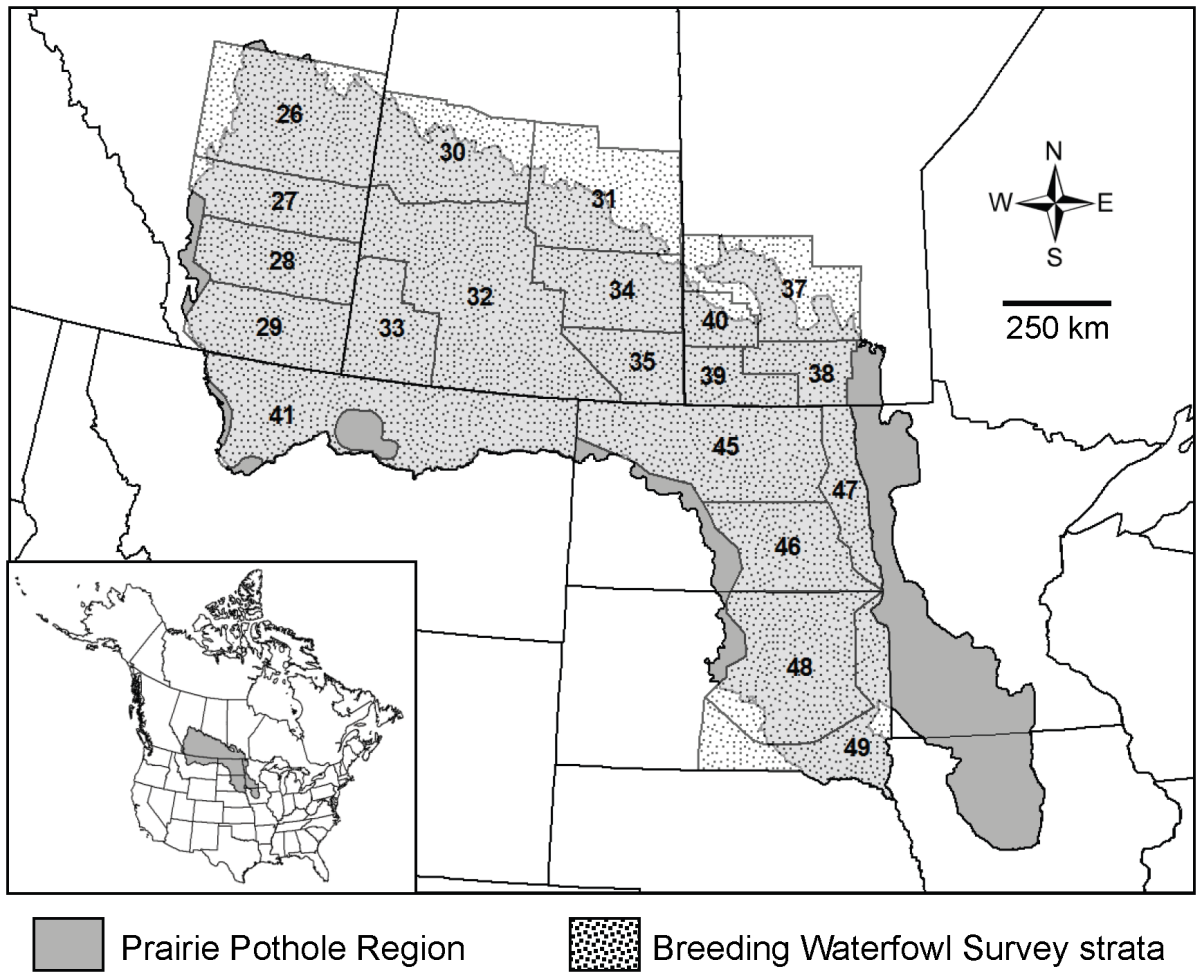
Location of North American Prairie Pothole Region. Twenty survey strata from the Waterfowl Breeding Population and Habitat Survey cover most of the land area, wetlands, and waterfowl resources of the Prairie Pothole Region. The survey strata extend from 96.5°W 42.5°N in the southeast to 114.8°W 54.0°N in the northwest.

The millions of small wetlands and associated grassland nesting habitat of the Prairie Pothole Region (PPR; Figure 1) make it the most important area for waterfowl production in North America [1], [2]. Consequently, the PPR is the focus of conservation programs that, in the United States alone, have permanently conserved >1.8 million ha of grasslands and wetlands (Table S1). Conservation efforts in the region are extensive and ongoing, as an additional 4.7 million ha of priority wetlands and grasslands must be protected to maintain waterfowl population goals in the US portion of the PPR as grasslands and wetlands continue to be converted to cropland [3], [4].

Prairie pothole wetlands are extremely productive because their shallow waters warm early in spring and their dynamic nature facilitates nutrient cycling and regeneration of vegetation and associated macro-invertebrates [5]–[7]. Waterfowl population size (Figure S1), nesting propensity, clutch size, and brood presence are positively related to wetland numbers [8]–[11], which are highly variable among years [12], [13]. Consequently, declines in the number and distribution of wetland basins containing water during the breeding season would reduce the ability of the PPR to attract and produce waterfowl.

Temperature and precipitation have generally increased in the PPR since the early to mid-1900s [14]–[16], although patterns differ between measures and among regions, time frames, and studies. Wetlands in the PPR may be particularly vulnerable to drying caused by increased temperatures associated with climate change because of their tenuous water balance and dynamic nature. Evaporation exceeds precipitation across much of the PPR, with conditions ranging from a positive water balance or lowest deficits in Iowa, where annual precipitation is highest, to highest deficits in northern Montana, southeastern Alberta, and southwestern Saskatchewan, although this gradient varies over space and time [17], [18]. Atmosphere-ocean general circulation models project that future temperatures and precipitation in the PPR will be higher than historic levels [19]. Based on simulation models developed to assess potential effects of these climate projections on prairie wetlands, the PPR is forecast to experience “increased drought conditions…under nearly all global circulation model scenarios” ([20]:864), with consequences for waterfowl predicted to be negative due to drier conditions in the western and central PPR but positive in the eastern PPR, which is expected to become wetter [20], [21]. Consequently, a significant recommendation from studies that have addressed potential effects of climate change on wetlands and waterfowl conservation in the PPR is to shift conservation efforts from the central and western PPR (primarily Saskatchewan, North Dakota, and South Dakota) to the eastern portion of the PPR (primarily Minnesota and Iowa) [20], [22]. However, in the short term, this shift may be costly: land protection and acquisition is more expensive in the eastern PPR, leading to less conservation “bang for the buck.” Additionally, conservation efforts in the PPR will face stronger competition from intensification of agriculture, which has been identified as “the most dominant factor affecting the distribution, abundance, and reproductive success of the region’s ducks” ([2]: 222; also [3], [23]). Land use is important to waterfowl, as the nesting success and brood presence of upland-nesting species, which comprise the majority of waterfowl in the PPR, are positively related to the amount of grass in the landscape [11], [24]–[27].

Given the magnitude of conservation efforts in the PPR (Table S1) and the potential impacts of climate change and other stressors on waterfowl and wetland-dependent species, recommendations to shift conservation efforts to the eastern PPR need to be carefully considered in terms of their short- and long-term consequences. Substantial uncertainty exists in our understanding of the complex interactions between climate, biological systems, socio-economic factors, and conservation costs. For example, how increased temperature and precipitation will affect wetland numbers and hydroperiod across the PPR is not easily predicted because of our limited understanding of hydrologic processes associated with widely varying precipitation/evapotranspiration ratios, geology, soil characteristics, and anthropogenic modifications [13], [28], [29].

We used 40 years of pond data collected during waterfowl surveys, along with broad-scale precipitation and temperature data, to investigate temporal and spatial trends in wetland numbers and hydroperiod and their relationship to precipitation and temperature trends in 20 waterfowl survey strata in the PPR. We sought to determine if trends in observed wetland numbers during this period were consistent with the decrease in wetland numbers predicted by published wetland models. In addition, we modeled wetland numbers as a function of precipitation and temperature to determine if broad-scale weather data could explain annual variation in wetland numbers. We discuss how the change in climate predicted for the PPR might interact with land use and other stressors to influence waterfowl ecology, and implications for the cost of effective conservation in the PPR. Because of considerable uncertainty and conflicting information related to climate change in the PPR, we also provide suggestions for reducing uncertainty relative to understanding the effects of climate change on waterfowl and conservation. We concentrate on waterfowl because they have been the primary focus for conservation and research related to climate change in the PPR, but our observations hold true for many other taxa. We focus our discussion on the US portion of the PPR, as conservation and agricultural programs and policies, as well as data availability, differ for Canada.

## Methods

### Trends in Wetland Numbers Over Time

Each May, biologists from the United States Fish and Wildlife Service and the Canadian Wildlife Service conduct aerial and ground surveys of wetlands and breeding waterfowl across major waterfowl-producing areas of the United States and Canada [30], [31]. Surveys are conducted along multiple transects within geographically distinct, non-overlapping strata, which were identified by delineating areas of similar habitat and duck densities. Aerial crews count the number of artificial (e.g., stockdams, dugouts) and natural ponds, which are then corrected for visibility using data collected by crews on the ground. Only ponds large enough to support waterfowl are counted. Using data from the US Fish and Wildlife Service Migratory Bird Data Center (https://migbirdapps.fws.gov/mbdc/databases/db_selection.asp), we analyzed numbers of May ponds for waterfowl survey strata entirely or largely within the PPR, an area that spans >1,800 km from central Alberta to southeastern South Dakota (*n* = 20; Figure 1). Because long-term populations of breeding waterfowl in the PPR are strongly related to May pond numbers, we assessed temporal trends in numbers of May ponds for each stratum by regressing annual May pond numbers against year. Given the highly variable nature of climate in the PPR, we focused on long-term trends and did not assess short-term cyclical or non-linear patterns in wetland numbers. We used data from 1974 to 2013, which includes all May pond survey data that were corrected for visibility [30]. The same methods were used throughout the survey period and we treat estimates as an index, rather than a census, of wetland numbers.

Because the number of May ponds differed substantially among strata, we were concerned that slope estimates might not be comparable among strata, i.e., the change in ponds per unit of time would be proportional to the number of ponds in each stratum. Therefore, we compared slope estimates for untransformed, standardized, and log–log regression models. Slopes for standardized and log–log models were strongly (*r* ^2^ = 0.64 and 0.63, respectively) and linearly related to slopes estimated using untransformed data, so we present results for models developed using untransformed data for ease of interpretation.

We also analyzed trends in July pond data, which were collected during the July Production Survey along the same transects as the May survey until 2003, when the July survey was discontinued. Unlike the May pond count, the July survey was not visibility corrected, but because transects were sampled by the same aerial crews as in May, data from the survey provide a relative index to pond conditions during nesting and brood-rearing periods. We estimated regression slopes of pond estimates on year for each stratum (*n* = 20) using July pond data from 1974 to 2003. In order to analyze the May pond counts together with July counts, we converted May pond counts into uncorrected counts by dividing by the visibility correction factor [30]. We also used the percent change in ponds from May to July, (July ponds – May ponds) × 100/May ponds, as an index of hydroperiod length to test the hypothesis that, in a changing climate, increased evapotranspiration caused by higher summer temperatures would shorten wetland hydroperiods, reducing the number of July ponds available for duck broods. Early drying of ponds to which waterfowl have been attracted has been suggested to be an ecological trap [21]. We used the percent change in pond counts, rather than the difference, to minimize differences in the magnitude of counts among strata. We regressed the percent change in ponds on year for the period 1974–2003 for each stratum in the PPR (*n* = 20).

### Climatic Trends and Factors Influencing Wetland Numbers

We acquired spatial data for monthly total rain gauge precipitation (mm) and monthly means of air temperature (degrees Celsius) interpolated from and cross-validated with weather station data [32], [33] from http://climate.geog.udel.edu/~climate/html_pages/download.htm. The spatial resolution of these data was 0.5 degree (approximately 50 km). To assign data to individual waterfowl survey segments, we resampled the dataset to a higher resolution (0.005 degree, or 1/100 of the original pixel dimensions), and calculated mean temperature and precipitation of pixels falling within the surveyed area of each segment (200 m on each side of the segment) using Environmental Systems Research Institute’s (ESRI) ArcGIS. For trend analysis and pond models the climate variables were the mean precipitation and temperature summarized over all segments within a stratum over the preceding year (May–April). As with the pond data, we used simple linear regression to assess trends in each stratum’s mean precipitation and temperature. Because precipitation and temperature data were only available for 1974–2010, time periods for these variables did not coincide completely with pond counts. We also used multiple linear regression to model May pond numbers as a function of temperature and precipitation for all strata combined. In the full model we included the previous year’s May pond numbers to account for temporal autocorrelation resulting from holdover in water conditions [34], [35], as well as a temperature*precipitation interaction term. Because of concerns about lagged dependent variables biasing other variables toward zero, we compared coefficients of variables in models with and without the previous year’s pond numbers. We standardized temperature and precipitation variables and the previous year’s pond numbers to facilitate comparison of model coefficients, and included the latitude and longitude of each stratum centroid as predictors to account for spatial patterns in pond counts that were not explained by spatial variation in climate. We discriminated among a set of models (full and reduced) using Akaike’s Information Criterion (AIC [36]) to identify the best model.

Because all data we used were collected remotely, no permits or guidelines for land access, handling of protected species, or animal husbandry were necessary. We conducted statistical analyses in the R environment and with Number Cruncher Statistical System 7.1 [37], [38].

## Results

### Trends in Wetland Numbers Over Time

**Figure 2:**
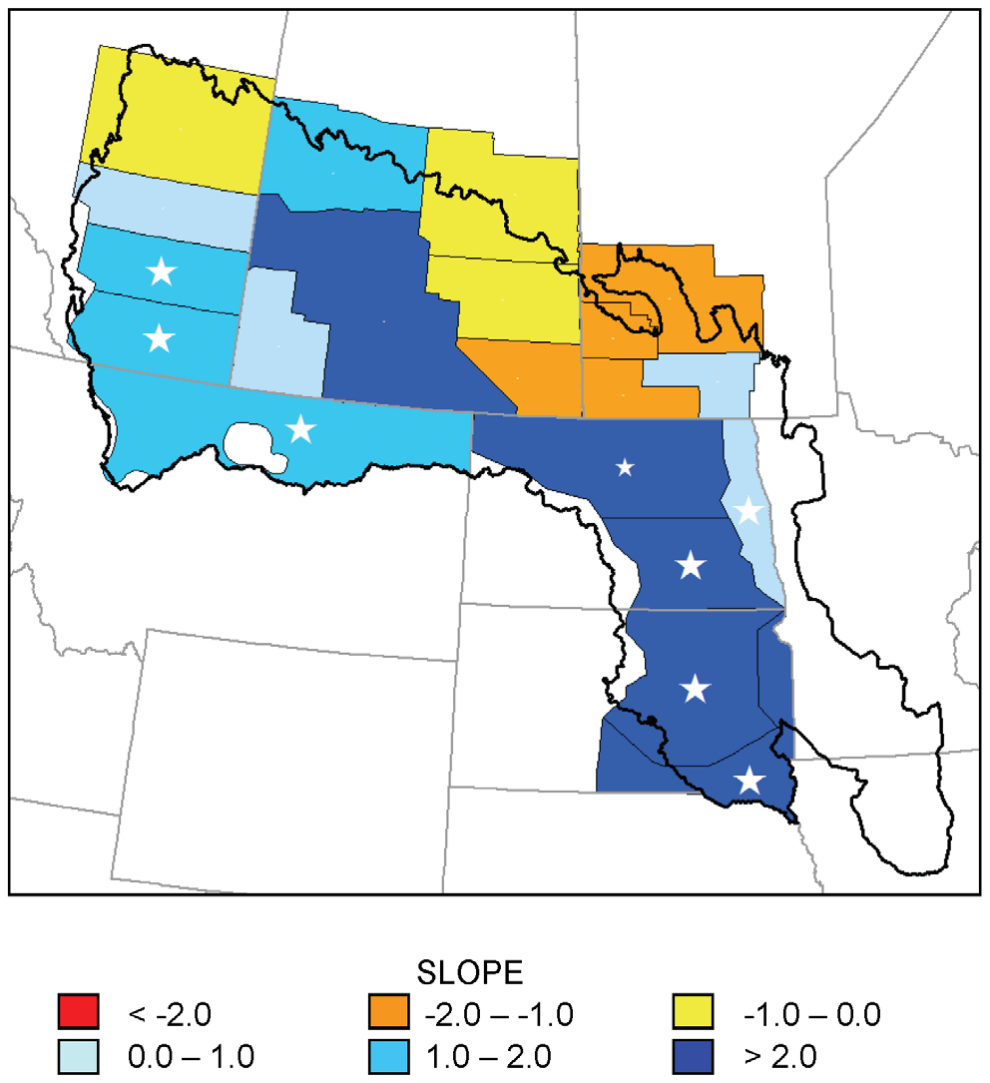
Trends in May pond numbers for 20 waterfowl survey strata, 1974–2013. Slope of regression models of May pond numbers (in thousands per year) in 20 strata of the Waterfowl Breeding Population and Habitat Survey as a function of year, 1974–2013. Large white stars indicate statistically significant trends (p<0.05); small white star indicates 0.05<p<0.1.

**Figure 3:**
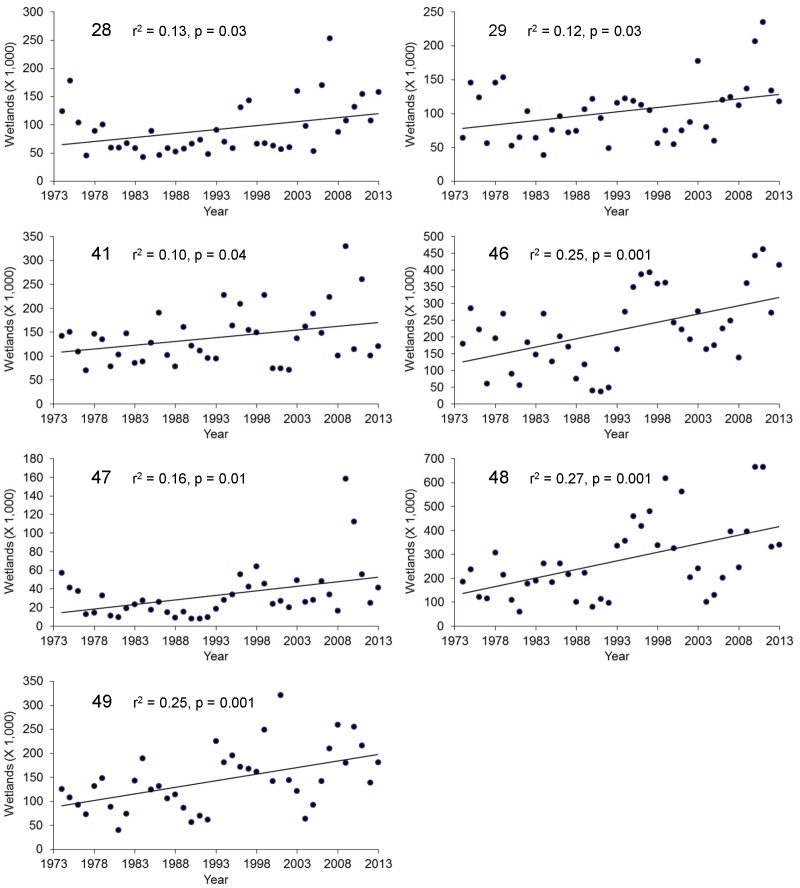
Trends in May pond numbers for seven waterfowl survey strata showing significant increases, 1974–2013. Number of May ponds (in thousands per year) increased significantly (p<0.05) in waterfowl survey strata 28, 29, 41, 46, 47, 48, and 49 of the Waterfowl Breeding Population and Habitat Survey, 1974–2013.

No strata showed statistically significant (p<0.05) declines in number of May ponds; however, seven strata showed significant increases (Figure 2). Inter-annual variation was high and cyclic patterns were sometimes evident; consequently, maximum model fit (coefficient of determination, *r* ^2^) for any stratum was 0.27 (Figure 3). The pattern in May pond numbers suggested a north–south gradient in trend, with strata in the southern portion of the PPR exhibiting significant increases in numbers of May ponds and strata in the northern portion of the PPR exhibiting non-significant increases or decreases (Figure 2).

**Figure 4:**
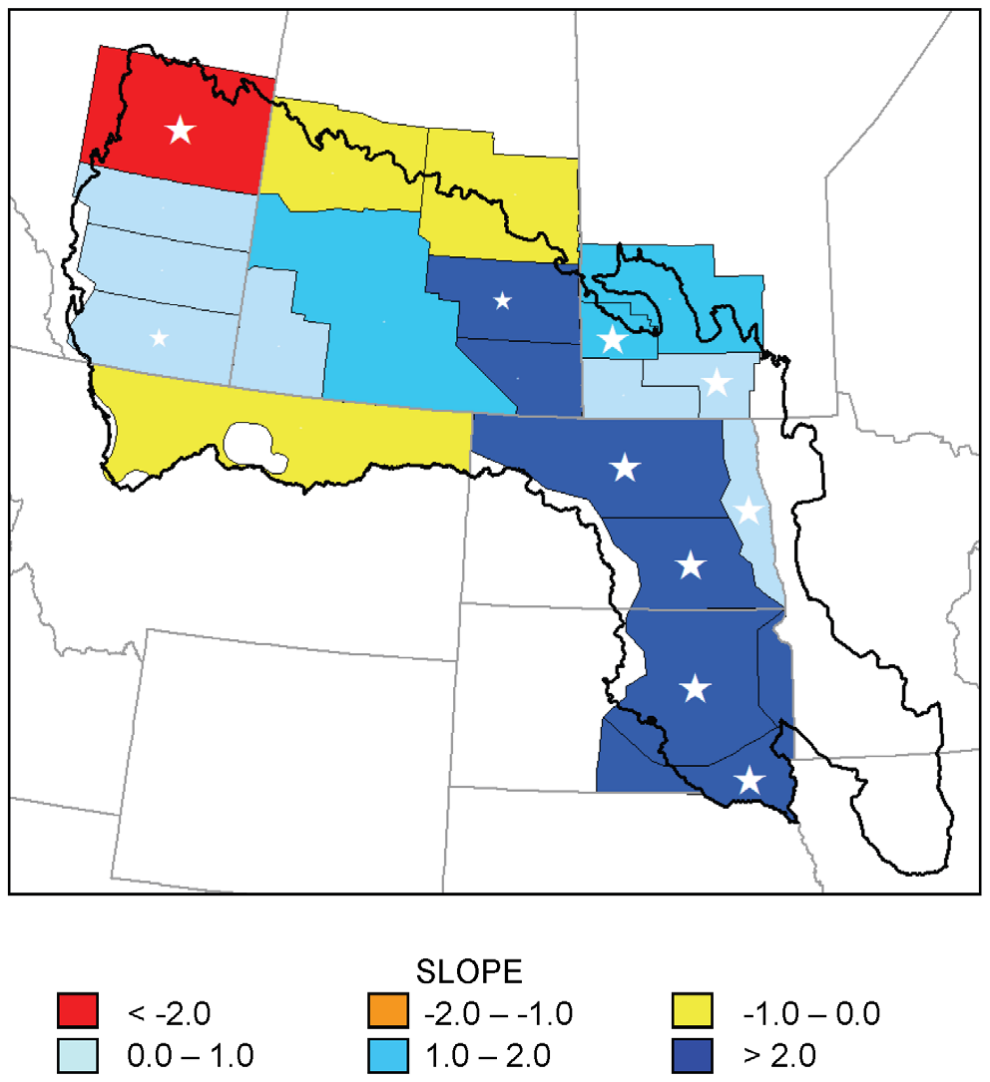
Trends in July pond numbers for 20 waterfowl survey strata, 1974–2003. Slope of regression models of July pond counts (in thousands per year) in 20 strata of the Waterfowl Breeding Population and Habitat Survey as a function of year, 1974–2003. Large white stars indicate statistically significant trends (p<0.05); small white stars indicate 0.05<p<0.1.

Seven strata (38, 40, 45, 46, 47, 48, 49) showed significant increasing trends in July ponds over time, and 1 stratum (26) showed a significant decreasing trend (Figure 4). The maximum *r* ^2^ of these regression models was 0.39, again due to substantial inter-annual variation. A spatial pattern similar to that of the May pond counts existed in the trend in July pond counts: increasing trends were primarily found in strata in the eastern and southern portion of the PPR, while the stratum with a significant decreasing trend was located in the northwest corner of the region.

**Figure 5:**
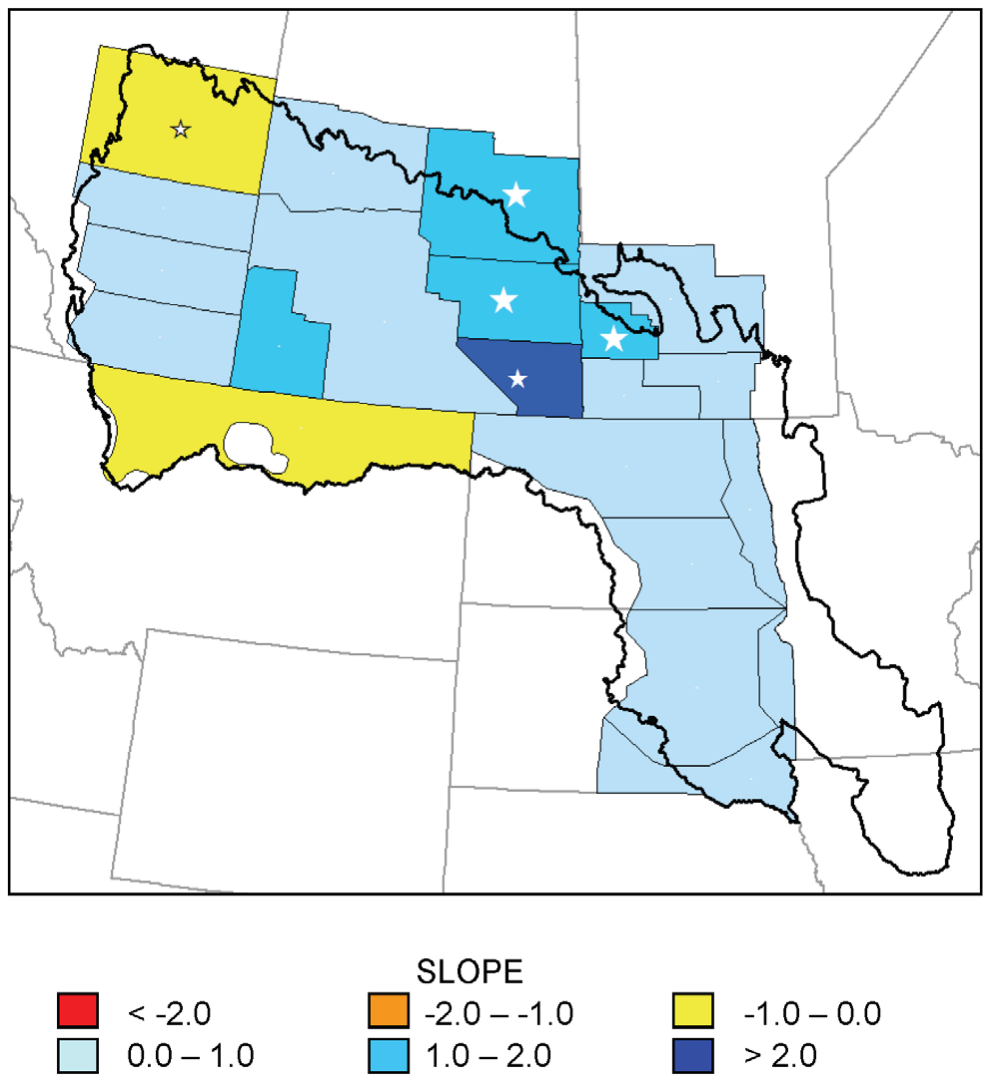
Trends in hydroperiod index for 20 waterfowl survey strata, 1974–2003. Slope of regression models of percent change in pond counts per year from May to July in 20 strata of the Waterfowl Breeding Population and Habitat Survey as a function of year (1974–2003). Large white stars indicate statistically significant trends (p<0.05); small white stars indicate 0.05<p<0.1.

The percent change in ponds from May to July exhibited a significant increasing trend in 3 strata located in the northcentral portion of the PPR (31, 34, and 40); all other trends were not statistically significant (Figure 5). The maximum *r* ^2^ for these models was 0.30.

### Climatic Trends and Factors Influencing Pond Numbers

**Figure 6:**
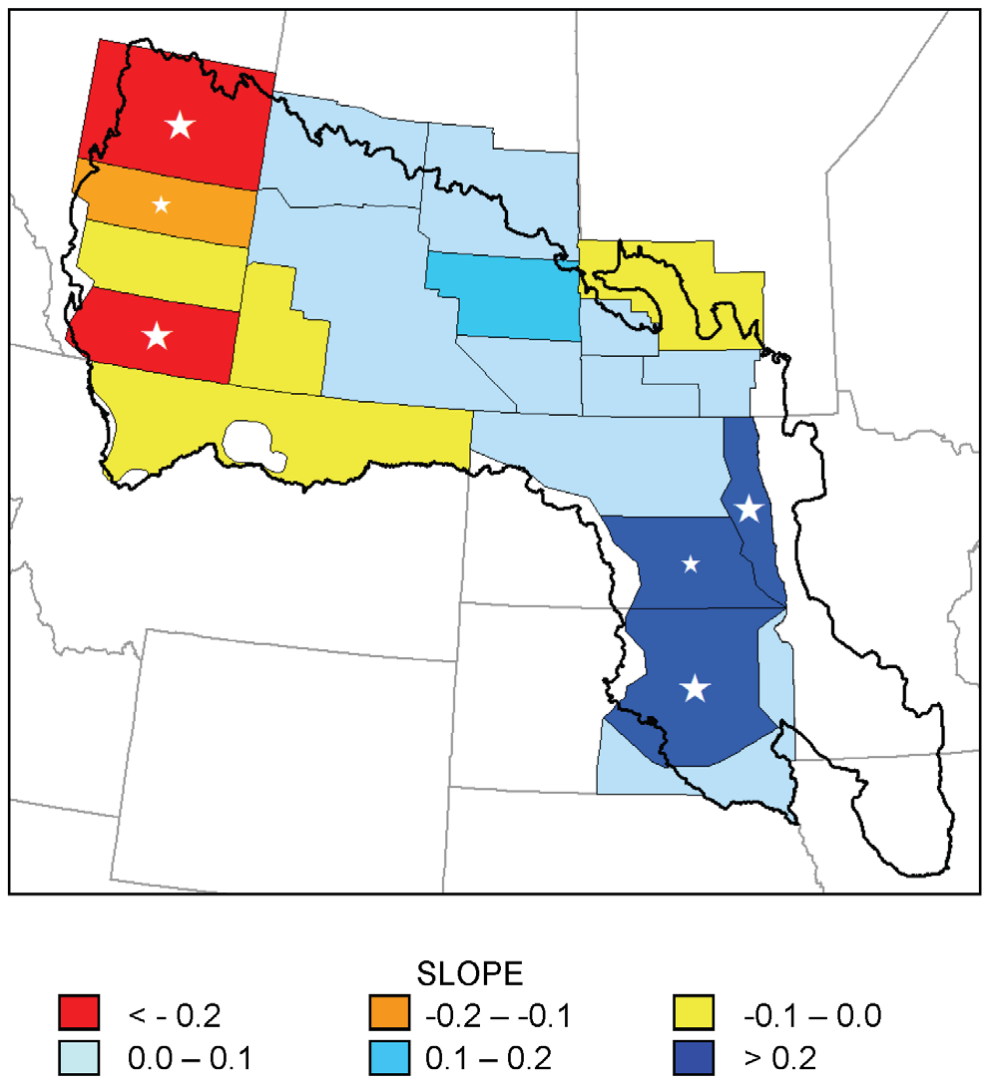
Trends in precipitation for 20 waterfowl survey strata, 1974–2010. Slope of trends in mean monthly total precipitation (mm per year) from 1974 to 2010 in 20 strata of the Waterfowl Breeding Population and Habitat Survey in the Prairie Pothole Region of the US and Canada. Data acquired from http://climate.geog.udel.edu/~climate/html_pages/download.htm [32]. White stars indicate statistically significant trends (p<0.05).

**Figure 7:**
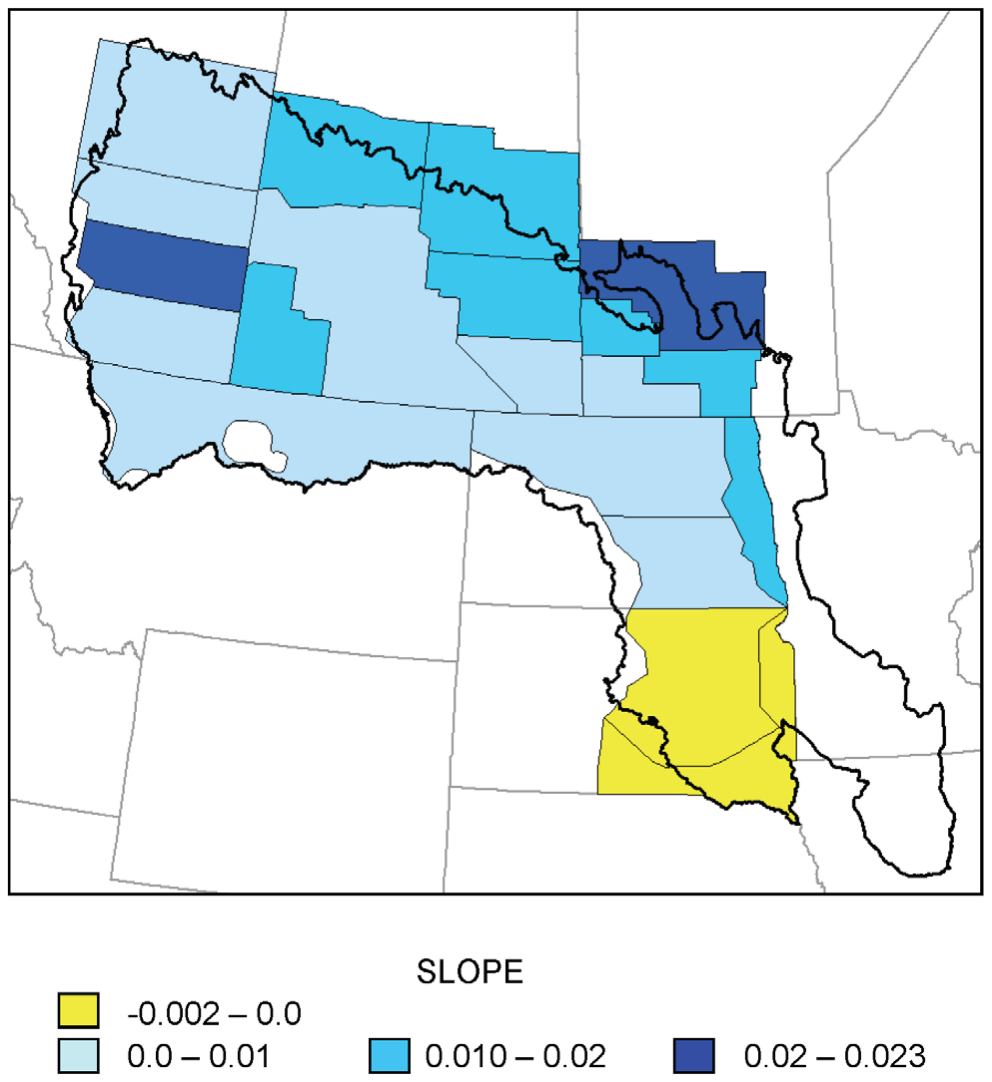
Trends in temperature for 20 waterfowl survey strata, 1974–2010. Slope of trends in mean monthly air temperature (degrees Celsius per year) from 1974 to 2010 in 20 strata of the Waterfowl Breeding Population and Habitat Survey in the Prairie Pothole Region of the US and Canada. Data acquired from http://climate.geog.udel.edu/~climate/html_pages/download.htm [33].

Mean monthly total precipitation for the period 1974–2010 showed statistically significant increasing trends in 2 strata in the southeastern portion of the PPR and significant decreasing trends in 2 strata in the western portion of the PPR (Figure 6). None of the strata showed significant trends in temperature during the same time period, although fitted slopes for 18 of 20 strata were positive (Figure 7).

Using data from all strata combined, the best model predicting pond numbers contained all the predictor variables except the ppt*temp interaction term. All coefficients in this model were statistically significant (P<0.0001; adjusted *r* ^2^ = 0.62). However, the full model (with the interaction term) also ranked highly (ΔAIC = 0.832), with model weight 39.7%, which suggested that the full model also had a relatively high probability of being the best approximating model. Therefore, we calculated model-averaged parameter estimates [36], resulting in a final model with the form:
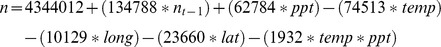
where *n* is the number of ponds; n*_t−1_* is the standardized previous year’s number of ponds; *ppt* is the standardized mean monthly accumulated precipitation for the previous year, measured in mm; *temp* is the standardized previous year’s mean air temperature, measured in degrees Celsius; *long* is the longitude of the stratum’s centroid; and *lat* is the latitude of the stratum’s centroid. All other models had ΔAIC values >137, so they were not considered competitive [36]. Although the lagged pond variable (previous year’s pond numbers) was a stronger predictor in the highest ranking model than the climate variables we included, the coefficients of the precipitation (52744) and temperature (−59009) variables in the full model (but with the previous year’s pond variable removed) were the same sign and slightly decreased in magnitude, indicating that these factors were contributing significant information in the current year’s pond numbers not explained by a temporal trend in pond numbers and were not biased toward zero by including the previous year’s pond numbers.

## Discussion

### Climate and Pond Trends

Increased numbers of May and July ponds over the 1974–2013 period (1974–2003 for July ponds) are not consistent with predictions of wetland drying [20], [21], [35], [39]. Trends in the change in pond counts from May to July suggest that, in at least the central portion of the PPR, the predicted shortening of hydroperiods due to increased evapotranspiration has not been occurring during this period. We detected increasing trends in precipitation in the southeastern PPR, which is consistent with the location of strata showing greatest increases in May and July pond numbers; the southeastern PPR was the only area to show a decreasing trend in temperature, although this trend, like increases in the rest of the PPR, was not statistically significant. Our results are consistent with wetland simulation models that suggest that increased precipitation can offset some effects of temperature on PPR wetlands [20], [34], [35]. Although our analysis used only 40 years of wetland data, which is too short a period to support or refute predictions of climate change, the trends we observed are certainly occurring within a time frame relevant to conservation planning.

Our results suggest that the primary conservation strategy in the US PPR of protecting waterfowl breeding habitat in areas of high waterfowl density is not presently jeopardized by drying of wetlands. Pond and climate data both suggest a spatial pattern of more water in the central PPR from southern South Dakota through Saskatchewan, although patterns varied with metric and time period. Of course, effects of climate change may be manifested in ways other than linear trends in numbers of ponds, such as future shifts in migration and breeding phenology of wetland-dependent birds or changes in the variation in wetness that typifies prairie potholes and makes them so productive. Nevertheless, trends in pond numbers indicate that, during the past 40 years, increases in the amount of precipitation have been sufficient to offset predicted effects of climate change on numbers of May and July ponds in much of the PPR. However, it is possible that areas of high conservation priority in the Dakotas might become drier in the future as climatic conditions change.

### Pond Models

Average daily temperature and total accumulated precipitation were statistically significant correlates of May pond numbers that, combined in a simple model with relatively coarse spatial and temporal resolution, explained 62% of variation in pond numbers. This suggests that, if current (i.e., past 37 years) observed trends in climate continue, pond numbers will respond in a similar manner in the near future. Of course, this does not imply that climate change is not happening in the PPR. Historical records suggest that the climate of the PPR is warming [14]–[16]; while we did not detect significant trends in temperature in the relatively short time period of our analysis, our pond model does predict that increasing temperature will result in fewer ponds. But in the short term, we propose that other stressors are having a much more pronounced impact on waterfowl populations in the PPR than climate-related drying of wetlands.

It is important to note that the short-term trends we report here, as well as the predicted long-term trends in climate change, do not capture the substantial cyclical and seasonal variability in wetland dynamics and climate in the PPR, which have a direct impact on waterfowl habitat quality. Coarse-scale climate data obscure much of the fine-scale variation in temperature and precipitation which influence wetland dynamics in the PPR. Variable hydrology may well be the defining characteristic of wetlands in the PPR because pond permanence and oscillating water levels are major drivers of ecological function of wetlands, influencing primary productivity, water salinity, nutrient cycling, invertebrate communities, composition and configuration of emergent vegetation, and wildlife [6]–[8], [40]–[43]. At some point, wetland productivity may decrease if the wetter, eastern portion of the PPR becomes wetter still and wetland water levels stay at unusually high levels. Wetlands that do not dry out also are more likely to harbor fish, which, whether native or introduced, can influence availability of aquatic invertebrates and ultimately reduce growth rates and survival of ducklings [44], [45].

### Shifting the Geographic Focus: Implications to Waterfowl Conservation in the US PPR

The current conservation paradigm in the PPR calls for the establishment of a diverse portfolio of land protection and restoration to account for temporal dynamics and spatial configuration of multiple habitat components needed by waterfowl [3], [46]. Changing the geographic focus of waterfowl conservation is more difficult than simply shifting the location of conservation efforts east to areas projected to have more water in the future. Waterfowl are necessarily associated with wetlands, but the majority of waterfowl in the PPR nest in adjacent uplands. Avian reproductive success is highly variable, but nesting success of many species of grassland-nesting birds, including waterfowl, typically increases with the amount of grass in the landscape due to reductions in predator densities associated with food crops and grassland fragmentation [26], [47]. Waterfowl conservation benefits are known to be higher in areas with large, contiguous grasslands relative to areas dominated by row crops [48]. Therefore, efforts to conserve waterfowl in the eastern “corn belt” portion of the PPR, an area predicted to be warmer and wetter due to climate change, will likely be less productive because less grass is present in the landscape relative to other portions of the US PPR [48, Figure S2]. In addition, the amount of grassland in the PPR could decrease further if increased precipitation and warmer temperatures lead to intensification of farming in the region [4,15,49–51, Figure S3].

A second problem with shifting waterfowl conservation efforts to the eastern PPR is that large numbers of wetland basins in the eastern PPR would need to be restored [52, figures S4 and S5], which would cost several times that of conserving existing wetlands. For example, the cost of a simple restoration such as plugging a wetland drainage ditch exceeds USD $988 per ha, and removal of sediment that accumulated as a consequence of tillage and erosion is approximately $2,500 per ha (Scott McLeod, USFWS, personal communication). Using these values and average cost of cropland in each state from 2012 (http://www.nass.usda.gov/Charts_and_Maps/Land_Values_and_Cash_Rents/crop_value_map.asp), the cost of purchasing land in fee title and restoring wetlands would be approximately $21,500 per ha in Iowa and $13,500 in Minnesota. Based on the purchase of a total of 24,450 ha of easements from 1 October 2011 to 30 September 2012, the mean cost per ha of perpetual wetland easements and perpetual grassland easements was $2,372 and $714, respectively, in North Dakota (Tammy Fairbanks, USFWS, personal communication) and $1,305 and $1,080 in South Dakota (William Mulvaney, USFWS, personal communication). Consequently, given the same resources, substantially less land could be conserved in the eastern portion of the PPR than in the western portion [53].

### Reducing Uncertainty Relative to Climate Change in the PPR

Given the extent and diversity of the PPR, discrepancies between long-term projections and short-term observed trends in pond numbers, and the complexity of conservation in the region, we present several issues that should be addressed to reduce uncertainty relative to both direct and indirect effects of climate change in the PPR.

First, we need to better understand wetland dynamics and their relationship to climate, in both short and long-term temporal scales. Inferences about how wetlands respond to changes in temperature and precipitation, and their interaction, should be based on data from samples representing a broad range of wetland types and conditions. Most of the long-term wetland data from which PPR hydrologic models have been developed come from wetlands in managed wildlife areas that are rarely subject to anthropogenic disturbance, such as tillage agriculture. This limits the inferences that can be made from these data, illustrating the observation of Felton et al. ([54]:2244) that “the propensity of ecologists to work in essentially unmodified ecosystems may fundamentally hamper our ability to make useful recommendation in a world that is experiencing global change.” An appropriate sampling framework is equally important to collecting data from which reliable inferences can be made. For example, 65% of the 3.25 million wetland basins identified by the National Wetlands Inventory in the PPR of North Dakota, South Dakota, Minnesota, and Iowa either touch or are surrounded by cultivated fields as identified through classified satellite imagery (Chuck Loesch and Rex Johnson, USFWS, unpublished data); data are needed to understand how these wetlands–which comprise such a large portion of the landscape–respond to future changes.

Second, collecting these data will require a new monitoring effort in the PPR, involving expansion of the extent or intensity of existing surveys, or concerted integration of multiple surveys and data sources. Open water is easily classified using remotely sensed, multi-spectral imagery, but the spatial resolution of remotely sensed data must be sufficiently fine to detect small wetlands, which dominate the PPR and are disproportionately important to waterfowl, as well as provide precise estimates of change in water area on small wetlands. In addition, correctly classifying water through emergent vegetation and collecting data during cloudy periods using remote sensing methods is problematic. Therefore, the sampling framework for future surveys must consider the full range of land use and physiographic variation across the PPR, with increased survey effort allocated to high-risk landscapes such as grasslands on fertile soils and wetlands in agricultural settings. Obtaining useful data may require new techniques or local collection, as classification error and thematic resolution of remotely sensed data will likely be insufficient to detect small changes typical of land surface change [55].

Finally, as conservationists working within a changing system, we need to embed this new monitoring effort within a context of adaptive decision making. Climate change will increase uncertainty in resource management [56]. Managers are faced with an optimization problem where the challenges of today must be confronted while considering the prospects of an uncertain tomorrow. Research needs to be focused on testing assumptions involved in our current decision making (in this case the allocation of conservation effort in regions of the PPR), investigating the interactions of climate and wetland dynamics in an anthropogenically modified system, and evaluating the relative threats of direct and indirect impacts of climate change.

Indirect effects of climate change that should be investigated include the relationship between increasing temperature and precipitation and land use intensification, specifically determining the rate of loss of native and tame grasslands, assessing changes in crop types, and determining the rate and type (e.g., tile versus ditch) of wetland loss. Other anthropogenic stressors that result from efforts to address climate change such as installation of wind turbines and planting of biofuel crops should also be monitored. Incorporating this information into our management framework allows us optimize the outcome of conservation decisions over both the short and long terms.

## Conclusions

In an assessment of the most important determinants of changes in biodiversity at the global scale over the next 100 years, land-use change was expected to have a greater effect than climate change in both terrestrial and freshwater ecosystems [57]. Several lines of evidence suggest that this may also be the case in the PPR, where agriculture is the dominant land use and tillage agriculture, which has demonstrated negative impacts on waterfowl populations, is increasing. Climate change may be a new reality in portions of the PPR as temperature, precipitation, and pond numbers have changed from historic levels. However, the threat of drying PPR wetlands may be a conservation “red herring” in the short term, if resultant shifts in conservation efforts benefit fewer ducks at increased expense while habitat loss and other impacts–which also are influenced by climate change–are ignored. If current trends continue, agricultural intensification exacerbated by climate change will likely further increase in portions of the PPR that presently harbor the greatest numbers of waterfowl. Therefore, assessments of the effects of climate change on waterfowl conservation must fully consider ecological, economic, and social realities along with the potential for climate-induced changes to determine the most effective places for conservation.

More information is needed before substantial changes are made to conservation strategies in the US PPR, but this should not be construed to mean inaction; instead of reacting to scenario-based models we believe a better approach is to develop competing hypotheses about how climate change is impacting waterfowl in the PPR, monitor wetland change, land use, and waterfowl response using well-designed surveys, and respond adaptively to those effects supported by data.

## Acknowledgments

We thank the many pilots and observers who have conducted May and July waterfowl surveys for many years; D. R. Hertel, R. R. Johnson, C. R. Loesch, and A. J. Ryba for providing or helping process spatial data; K. Hunter and S. Hutchcroft for providing data on protected lands; and E. Babij, B. Bond-Lamberty, G. Liu, R. R. Johnson, W. A. Meeks, K. D. Richkus, E. D. Silverman, C. L. Stemler, J. A. Walker, and one anonymous reviewer for helpful comments on earlier drafts of the manuscript. The findings and conclusions in this article are those of the authors and do not necessarily represent the views of the US Fish and Wildlife Service.

## Supporting Information

Figure S1 **Waterfowl populations are strongly related to wetland numbers.** Annual population estimates for seven species of breeding dabbling ducks from the Waterfowl Breeding Population and Habitat Survey were strongly related to annual estimates of May pond numbers in the Canadian (upper) and US (lower) portions of the Prairie Pothole Region, 1974–2013. Data acquired from the US Fish and Wildlife Service Migratory Bird Data Center (https://migbirdapps.fws.gov/mbdc/databases/db_selection.asp).
(TIFF)

Figure S2 **Grassland in the US Prairie Pothole Region.** The amount of grass in the landscape, which is positively associated with waterfowl nesting success, is generally correlated with a precipitation gradient that allows more intensive agriculture in the southeastern portion of the PPR. Grassland was defined as herbaceous cover, hay, and pasture cover classes identified by the 2006 National Landcover Database [58], available at www.mrlc.gov. We restricted the landscape portion of our analysis to the US because comparable data were not available for the PPR of Canada.
(TIF)

Figure S3 **Changes in area of corn and soybeans harvested for grain, 1997–2007.** Area of corn (Zea mays, upper) and soybeans (Glycine max, lower) harvested for grain increased substantially in the Prairie Pothole Region 1997–2007 relative to the rest of the conterminous United States, which showed decreases or small increases in area harvested per county. Data available at http://www.agcensus.usda.gov/index.php. We restricted the landscape portion of our analysis to the US because comparable data were not available for the PPR of Canada.
(TIF)

Figure S4 **Wetland density in the US Prairie Pothole Region.** Wetland density, which is positively associated with waterfowl settling and negatively influenced by agricultural development, is lowest in the southeastern portion of the US Prairie Pothole Region and is generally highest in central North Dakota and South Dakota. Wetland density derived from National Wetlands Inventory data [59] (available at http://www.fws.gov/wetlands/Data/DataDownload.html) processed to basins [60]. We restricted the landscape portion of our analysis to the US because comparable data were not available for the PPR of Canada.
(TIF)

Figure S5 **Density and distribution of five priority duck species.** Modeled density and distribution of five priority species of waterfowl (mallard [Anas platyrhynchos], gadwall [A. strepera], northern pintail [A. acuta], northern shoveler [A. clypeata], and blue-winged teal [A. discors]) in the US PPR are strongly related to wetland density. Figure derived following methodology of Reynolds et al. [27] and unpublished data courtesy of Sean P. Fields and Rex R. Johnson, USFWS.
(TIF)

Table S1 **Hectares of land protected in fee title on National Wildlife Refuges (NWR) and Waterfowl Production Areas (WPA) or by perpetual wetland or grassland easement held by the US Fish and Wildlife Service in the Prairie Pothole Region of Montana, North Dakota, South Dakota, Minnesota, and Iowa.**
(DOCX)
