# Supplementary figures and images for: Waterfowl Conservation in the US Prairie Pothole Region: Confronting the Complexities of Climate Change
Source: PLoS One. 2014 Jun 17;9(6):e100034. doi: 10.1371/journal.pone.0100034 (PMC4061047; doi:10.1371/journal.pone.0100034)

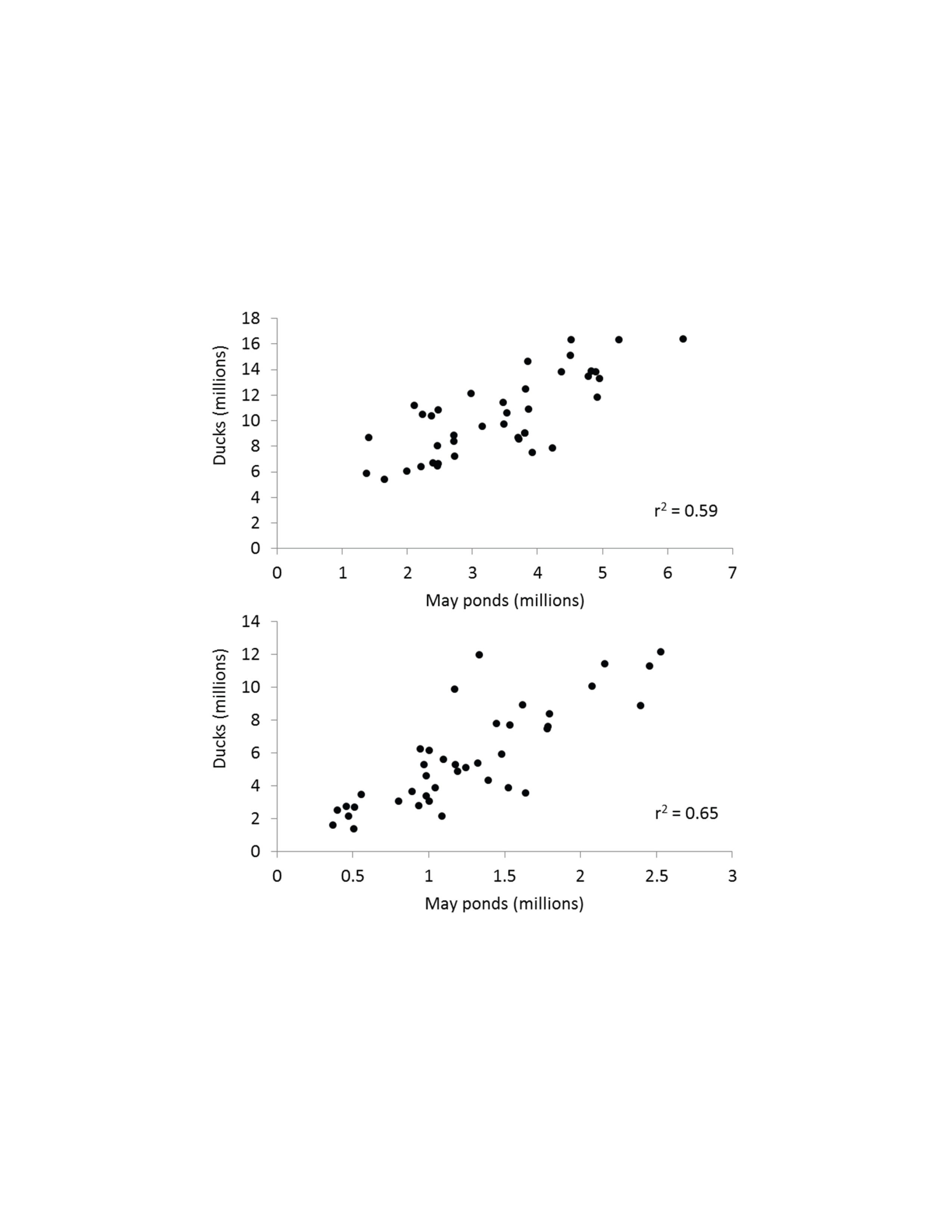

Supplement: Figure S1 — Waterfowl populations are strongly related to wetland numbers. Annual population estimates for seven species of breeding dabbling ducks from the Waterfowl Breeding Population and Habitat Survey were strongly related to annual estimates of May pond numbers in the Canadian (upper) and US (lower) portions of the Prairie Pothole Region, 1974–2013. Data acquired from the US Fish and Wildlife Service Migratory Bird Data Center (https://migbirdapps.fws.gov/mbdc/databases/db_selection.asp). (TIFF) [file pone.0100034.s001.tiff]

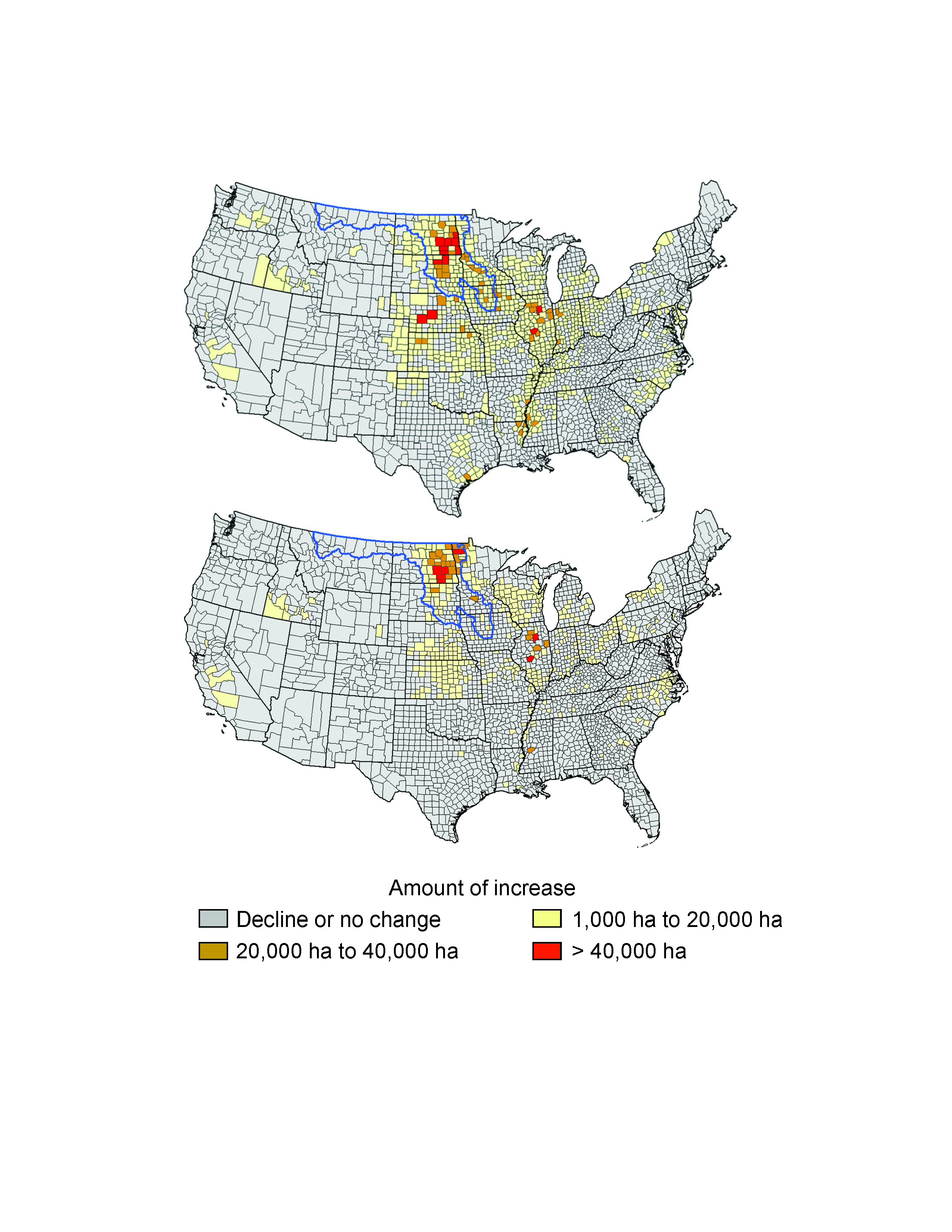

Supplement: Figure S3 — Changes in area of corn and soybeans harvested for grain, 1997–2007. Area of corn (Zea mays, upper) and soybeans (Glycine max, lower) harvested for grain increased substantially in the Prairie Pothole Region 1997–2007 relative to the rest of the conterminous United States, which showed decreases or small increases in area harvested per county. Data available at http://www.agcensus.usda.gov/index.php. We restricted the landscape portion of our analysis to the US because comparable data were not available for the PPR of Canada. (TIF) [file pone.0100034.s003.tif]
