# Supplementary material for: Waterfowl Conservation in the US Prairie Pothole Region: Confronting the Complexities of Climate Change
Source: PLoS One. 2014 Jun 17;9(6):e100034. doi: 10.1371/journal.pone.0100034 (PMC4061047; doi:10.1371/journal.pone.0100034)
Supplement: Table S1 — Hectares of land protected in fee title on National Wildlife Refuges (NWR) and Waterfowl Production Areas (WPA) or by perpetual wetland or grassland easement held by the US Fish and Wildlife Service in the Prairie Pothole Region of Montana, North Dakota, South Dakota, Minnesota, and Iowa. (DOCX) [file pone.0100034.s006.docx]

| State | NWR | WPA | Wetland Easement | Grassland Easement | Total |
| --- | --- | --- | --- | --- | --- |
| Montana | 210,900 | 12,800 | 11,400 | 36,200 | 290,800* |
| North Dakota | 86,400 | 116,900 | 354,800 | 163,700 | 721,800 |
| South Dakota | 17,900 | 65,600 | 214,000 | 333,900 | 631,400 |
| Minnesota | 54,700 | 79,100 | 26,300 | 9,300 | 169,400 |
| Iowa | 3,500 | 10,100 | 200 | <100 | 13,800 |
| Total | 373,400 | 284,500 | 606,700 | 543,100 | 1,827,200 |

*Total for Montana includes 19,500 ha of non-development easements which includes varying amounts of grasslands and wetlands.
